# Age-related changes in intraventricular kinetic energy: a physiological or pathological adaptation?

**DOI:** 10.1152/ajpheart.00075.2015

**Published:** 2016-01-08

**Authors:** James Wong, Radomir Chabiniok, Adelaide deVecchi, Nathalie Dedieu, Eva Sammut, Tobias Schaeffter, Reza Razavi

**Affiliations:** ^1^Department of Imaging Sciences, Kings College London, St Thomas' Hospital, London, United Kingdom;; ^2^Inria and Paris-Saclay University, Palaiseau, France

**Keywords:** four-dimensional phase-contrast magnetic resonance imaging, cardiovascular magnetic resonance, energy, cardiac function, heart failure

## Abstract

Aging has important deleterious effects on the cardiovascular system. We sought to compare intraventricular kinetic energy (KE) in healthy subjects of varying ages with subjects with ventricular dysfunction to understand if changes in energetic momentum may predispose individuals to heart failure. Four-dimensional flow MRI was acquired in 35 healthy subjects (age: 1–67 yr) and 10 patients with left ventricular (LV) dysfunction (age: 28–79 yr). Healthy subjects were divided into age quartiles (1st quartile: <16 yr, 2nd quartile: 17–32 yr, 3rd quartile: 33–48 yr, and 4th quartile: 49–64 yr). KE was measured in the LV throughout the cardiac cycle and indexed to ventricular volume. In healthy subjects, two large peaks corresponding to systole and early diastole occurred during the cardiac cycle. A third smaller peak was seen during late diastole in eight adults. Systolic KE (*P* = 0.182) and ejection fraction (*P* = 0.921) were preserved through all age groups. Older adults showed a lower early peak diastolic KE compared with children (*P* < 0.0001) and young adults (*P* = 0.025). Subjects with LV dysfunction had reduced ejection fraction (*P* < 0.001) and compared with older healthy adults exhibited a similar early peak diastolic KE (*P* = 0.142) but with the addition of an elevated KE in diastasis (*P* = 0.029). In healthy individuals, peak diastolic KE progressively decreases with age, whereas systolic peaks remain constant. Peak diastolic KE in the oldest subjects is comparable to those with LV dysfunction. Unique age-related changes in ventricular diastolic energetics might be physiological or herald subclinical pathology.

## Abstract (precis)

*Measuring intracardiac kinetic energy using four-dimensionl flow cardiac magnetic resonance provides important information on the decline in the early diastolic kinetic energy of blood with aging. The decline is comparable with that seen in those with heart failure and may be a marker of cardiac function*.

## NEW & NOTEWORTHY

*Measuring intracardiac kinetic energy using four-dimensionl flow cardiac magnetic resonance provides important information on the decline in the early diastolic kinetic energy of blood with aging. The decline is comparable with that seen in those with heart failure and may be a marker of cardiac function*.

during healthy aging, the left ventricle (LV) and cardiovascular system are known to undergo adverse changes including increased stiffness of blood vessels, ventricular remodeling, and decreased ventricular compliance (1). Aging is associated with a higher risk of cardiovascular conditions such as hypertensive disease, atrial fibrillation, and heart failure (23–25). Fifty percent of all diagnoses of heart failure occur in those aged over 70 yr. The changes in ventricular morphology and related function that occur with healthy aging may lower the threshold for developing heart failure (37). This, alongside the increasing global population aged 65 yr or older, underlines the need understand the spectrum of age-related changes that occur in the cardiovascular system.

There has been recent interest in the use of four-dimensional (4-D) phase-contrast (PC) flow MRI to noninvasively measure intracardiac kinetic energy (KE) (5–11). 4-D flow MRI provides a detailed overview of the velocity of blood in three directions, helping to capture the intricacies of blood flow within the heart. This detail is not adequately captured by two-dimensional (2-D) Doppler echocardiography or 2-D PC flow MRI, which are limited to a single imaging plane and measure velocities in only one direction. Early work qualifying ventricular blood flow characteristics in a small group of healthy subjects of different ages has shown that the number of blood vortexes decreases with age (10). This affects the flux of energy, and quantifying such changes may provide a better understanding of this process. One method may be to use 4-D flow MRI to measure the KE of blood during the cardiac cycle. The KE of blood represents a small yet essential component of work performed by the heart (31) as a measure of the inertia of blood (38, 41). Distinct intracardiac KE patterns exist between the LV and right ventricle (RV) (5), and unique alterations representing a loss of the momentum of blood are apparent in those in heart failure (7). The compliance and recoil of the LV play an important role in efficient ventricular energetic filling. The diastolic KE of blood produced as a consequence of active relaxation of the ventricle is important and in healthy LVs is higher than the KE of blood in systole (5). However, falling compliance occurs both as an age-related adaptation (12) and in those with LV dysfunction. This may have an impact on the KE profile of the ventricle and its ability to conserve the momentum of blood. Measuring KE in healthy subjects of different ages and comparing these values with patients with established heart failure, such as in dilated cardiomyopathy, may help determine whether the pattern seen in aging represents a spectrum of physiological decline or a distinct pathological process. We hypothesized that with increasing age, intracardiac KE would show significant differences most apparent in diastole. To test this hypothesis, we measured intracardiac KE in a broad age group of healthy individuals and a group of patients with LV dysfunction as a positive control.

## MATERIALS AND METHODS

### 

#### Study design.

Data were collected prospectively on 35 adults and children (27 adults and 8 children) and 10 patients with LV dysfunction. Patients with LV dysfunction were defined by the signs and symptoms of heart failure with additional echocardiographic evidence of ventricular dilatation and systolic dysfunction as measured by depressed ejection fraction (EF). Patients with LV dysfunction were age and sex matched to a subsection of the healthy volunteer group.

All healthy subjects were without cardiovascular disease, in sinus rhythm, with normal ECG and normal blood pressure. As per institutional preference, subjects under the age of 10 yr were scanned under general anesthesia using low-dose inhaled sevofluorane with remifentanyl while maintaining normocarbia. Subjects with LV dysfunction were clinically New York Heart Association class I or II. All subjects underwent cardiac MRI on a 1.5-T scanner (Achieva, Philips Healthcare, Best, The Netherlands). The local research ethics committee approved the study design, and all adult participants gave written consent (10/H0802/65). The local research and ethics committee approved that children undergoing a clinically indicated MRI scan (anesthetized or unanesthetized) could undergo an additional 15 min of scanning for research purposes (09/HO802/78). The attending medical professional identified children with structurally and functionally normal hearts who were felt suitable for inclusion into the study. The family was provided with verbal and written information about the study and was allowed to consider whether they wished to participate and to seek further information if necessary. A signed consent form was completed for all experimetns. Assent was taken from younger children where they wished and was sought in all children who were deemed competent.

#### Cine imaging.

Retrospectively ECG-gated balanced steady-state free precession cine four-chamber views and short-axis stacks were performed with the short axis planned parallel to the mitral valve plane. Images were acquired during end-expiratory breath holds covering the apex to base. Typical imaging parameters were as follows: repetition time (TR), 3.0–3.6 ms; echo time (TE), 1.5–1.8 ms; parallel imaging factor (using SENSE), 2; flip angle, 60°; field of view, 200–400 mm; slice thickness, 6–10 mm depending on patient size; in-plane resolution, 1.3–2.0 mm; acquired temporal resolution, 30–40 phases, and breath-hold duration, 5–7 s/slice with 10–14 slices to cover the whole heart including the proximal aorta. A 15-mm respiratory gating window was used to ensure that breath holds were consistent between slices to reduce spatial misalignment. Analysis of volumetric data was performed using a Viewforum workstation (Viewforum, release 2.0, Philips Healthcare). Segmentation of the ventricular cavity involved manual tracing of the endocardial contour for each slice at end systole and end diastole (27). Trabeculations and papillary muscles were carefully excluded. The position of the atrioventricular valve was taken into account and confirmed by linking the short-axis stack to the four-chamber view of the ventricle.

#### 2-D PC flow imaging.

A free-breathing retrospectively ECG triggered 2-D PC scan orthogonal to the ascending aorta at the level of the right pulmonary artery was acquired with three signal averages, a spatial resolution of 1.5 × 1.5 × 6 mm, and an acquired temporal resolution of 30 phases. The peak velocity of flow in the aorta was used to target the velocity encoding range of the 4-D flow PC scan.

#### 4-D PC flow.

A free-breathing prospectively ECG triggered 4-D flow whole heart MRI sequence was acquired using a targeted velocity encoding based on 2-D PC aortic peak velocity. The mean field of view was 300 × 70 × 150 mm, with the following spatial resolution specific to the size of the patient: small children (<20 kg), 2.0-mm isotropic voxels; large children and adults (20–90 kg), 2.5-mm isotropic voxels; and large adults (>90 kg), 3-mm isotropic voxels. The number of phases was adjusted to between 24 and 32 phases to acquire a temporal resolution of <35 ms. Other imaging parameters included the following: TR, 3.8 ms; TE, 2.4 ms; flip angle, 5; acceleration kt+, 8; and bandwidth, 500 Hz. Respiratory gating for motion correction was applied giving a nominal scan time of 5–7 min (14, 15). Data were reconstructed using an in-house implemented kt-principal component analysis method (22, 29) and subsequently corrected for background errors. The analysis of flow data was performed using proprietary software (GTFlow, GyroTools, Zurich, Switzerland). Balanced cine steady-state free precession images were used to help segment the motion of the mitral valve annulus throughout the cardiac cycle on the 4-D PC imaging. This allowed us to measure peak through-plane inflow velocities into the LV (the mitral E wave) and compare them with changes in diastolic KE.

#### Calculation of KE.

Initially, the endocardial border of the LV was manually segmented in the first timeframe from the short-axis cine stack balanced steady-state free precession images using CardioViz3D software (39). This allowed the generation of endocardial surfaces and a separately labeled mask image. LV motion, as seen in the cine data, was tracked by an image registration based method (34, 35) using the “Image Registration Toolkit” (IXICO) to create displacement fields. The displacement fields were used to morph the systemic ventricular myocardial mask, creating a 4-D ventricular mask.

Particles from the 4-D flow sequence were seeded within the mask at a density equivalent to the voxel size of the 4-D flow images for each timeframe and the 4-D PC data used to measure the velocity.

KE was calculated by taking the instantaneous velocity magnitude of a particle and applying the following formula: KE = ½ mass × velocity^2^. The mass of blood was derived from multiplying the mean density of blood (1,060 g/mm^3^) by the voxel volume represented by each particle.

**Fig. 1.**
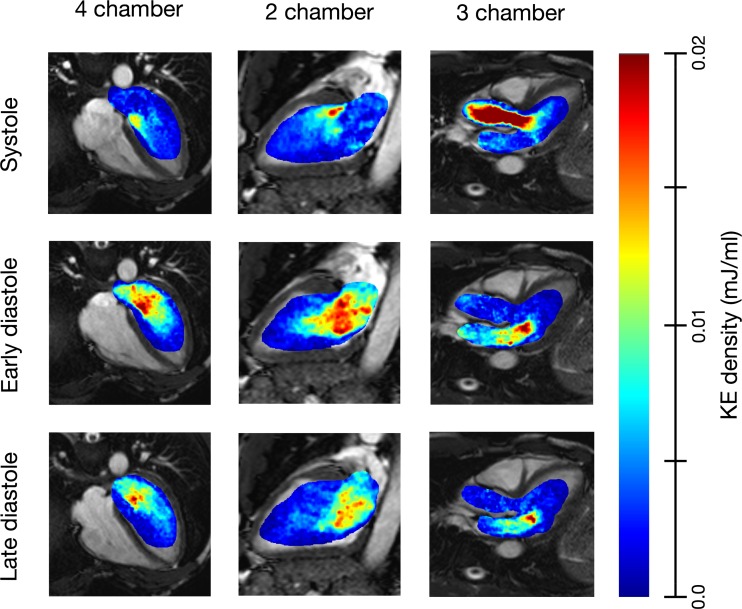
Illustrative example of two-, three-, and four-chamber views of a healthy heart demonstrating kinetic energy (KE) density during different portions of the cardiac cycle.

To allow comparison between hearts of different sizes, KE values were expressed as KE density based on the instantaneous volume blood present in the ventricle at that phase of the cardiac cycle. The resultant parameter was expressed in the form of energy per milliliter of blood (in mJ/ml). KE values were expressed as a fraction of the R-R interval. KE was measured throughout the cardiac cycle. KE in different parts of the cardiac cycle is shown in Fig. 1 for a healthy LV.

#### Statistical analysis.

Statistical analysis was performed using SPSS (version 22). Healthy subjects were divided into age quartiles (1st quartile: <16 yr, 2nd quartile: 17–32 yr, 3rd quartile: 33–48 yr, and 4th quartile: 49–64 yr). Unpaired *t*-tests were used for intergroup comparisons in cohorts containing only two subgroups (e.g., 4th age quartile vs. LV dysfunction). Paired *t*-tests were used for comparison of KE in the age- and sex-matched subjects. For cohorts with four subgroups (e.g., age quartiles), one-way ANOVA with Bonferroni-adjusted, post hoc *t*-tests were used for the majority of variables, provided the assumptions for ANOVA were met (normal distribution and equality of variance). *P* values of <0.05 were considered significant. Allometric cardiac growth occurs with age, so linear indexing to body surface area is permissible only between fully grown adults of different sizes (3, 33). Therefore, KE was indexed to the volume of blood within the ventricle at each recorded time point and expressed as a proportion of the R-R ECG interval to allow comparison between subjects of differing sizes, heart rates (HRs), and function.

## RESULTS

Thirty-five individuals ranging in age from 1 to 67 yr were recruited and divided into four age quartiles: 8 children (<16 yr), 11 young adults (17–32 yr), 11 middle-aged adults (33–48 yr), and 5 older adults (49–64 yr). The reasons for the MRI referrals in children were as follows: exclusion of vascular ring (*n* = 3), assessment for tortuosity of the head and neck vessels (*n* = 2), assessment for arrhythmogenic RV cardiomyopathy (*n* = 2), and exclusion of partial anomalous venous drainage (*n* = 1). All children included in the study were confirmed to have structurally and functionally normal hearts by a cardiologist experienced in reporting cardiac magnetic resonance (CMR). Ten patients with compensated LV dysfunction were used as positive controls (age: 28–79 yr).

Table 1 shows the patient demographics. As expected, children had a higher HR compared with the older age quartiles, although mean HR and blood pressure were matched between the healthy group and those with LV dysfunction. Table 2 shows MRI-derived parameters of cardiac function. An increase in ventricle size was evident between childhood and adulthood, consistent with allometric ventricular growth (4, 19, 36); however, EF was equal at all ages. Indexed wall mass increased between childhood and adulthood (*P* = 0.007). The LV dysfunction group had reduced EF (*P* < 0.001) and dilated hearts (*P* < 0.034) compared with the healthy older adult group.

**Table 1.** Subject demographics

Parameter	Healthy Group	LV Dysfunction Group	*P* Value
Sex			
Male subjects	19	5	
Female subjects	16	5	
Race			
Caucasian	31	9	
Asian	4	1	
Mean age ± SD, yr	29 ± 13	51 ± 15	<0.001
Mean BSA, m^2^ (range)	1.7 (0.5–2.2)	1.9 (1.3–2.5)	0.061
Mean heart rate ± SD, beats/min	70 ± 19	67 ± 14	0.497
1st quartile (<16 yr, *n* = 8)	104 ± 23		
2nd quartile (17–32 yr, *n* = 11)	65 ± 14	60	
3rd quartile (33–48 yr, *n* = 10)	63 ± 8	68	
4th quartile (49–64 yr, *n* = 6)	61 ± 7	65 ± 12	
Mean blood pressure ± SD, mmHg			
Systolic	118 ± 10	128 ± 27	0.123
Diastolic	74 ± 10	76 ± 10	0.667
Medication			
Angiotensin receptor blocker/angiotensin-converting enzyme inhibitor	0	10	
β-Blocker	0	10	
CC antagonist	0	3	
Diuretic	0	5	
Aspirin	0	4	
Statin	0	2	
Warfarin	0	1	

LV, left ventricular.

**Table 2.** MRI-derived LV volumetrics and function

	Indexed End-Diastolic Volume, ml/m^2^	Indexed Stroke Volume, ml/m^2^	Ejection Fraction, %	Indexed Wall Mass, g/m^2^
LV 1st quartile (<16 yr, *n* = 8)	68.9 (±9.9)†	41.5 (±7.2)†	59.0 (±3.3)	48.8 (±5.5)*
LV 2nd quartile (17–32 yr, *n* = 11)	84.9 (±12.0)	50.8 (±6.6)	60.1 (±5.2)	64.6 (±17.3)
LV 3rd quartile (33–48 yr, *n* = 11)	81.2 (±12.6)	49.9 (±8.4)	61.7 (±6.4)	61.0 (±13.5)
LV 4th quartile (49–64 yr, *n* = 5)	90.2 (±8.9)	52.3 (±4.2)	58.1 (±3.9)	78.1 (±14.9)
*P* value by ANOVA	0.009	0.024	0.516	0.005
LV dysfunction	105.4 (±25.7)*	40.1 (±10.9)*	40.0 (±11.7)*	73.0 (±16.1)
*P* value (older adults vs. LV dysfunction)	0.04	0.03	0.0001	0.475

*P* values compared with older adults are shown.

* *P* < 0.01; †*P* < 0.05.

**Fig. 2.**
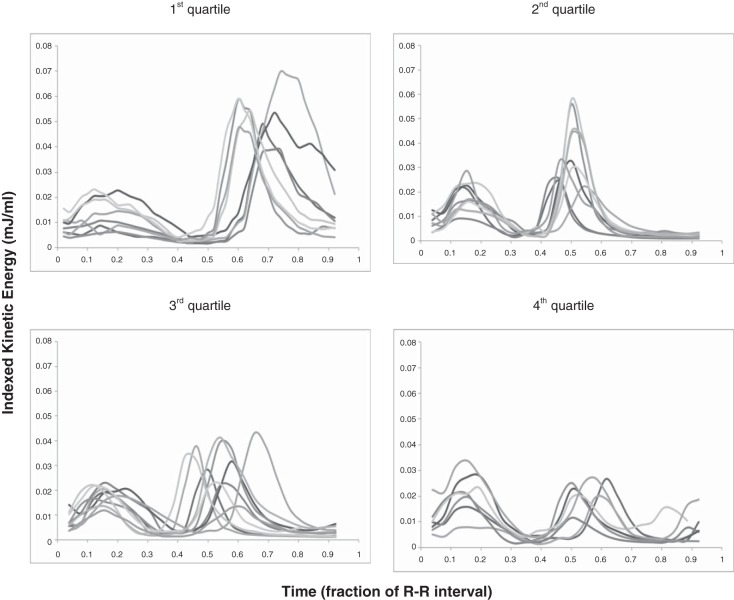
KE in the left ventricle (LV) according to age. These images show KE indexed to the ventricular volume at each time point during the cardiac cycle. Indexing KE to the volume of blood at that time allowed comparison between subjects of different sizes. The cardiac cycle was divided into fractions of the R-R interval. *Top left*: 1st age quartile; *top right*: 2nd age quartile; *bottom left*: 3rd age quartile; *bottom right*: 4th age quartile.

**Fig. 3.**
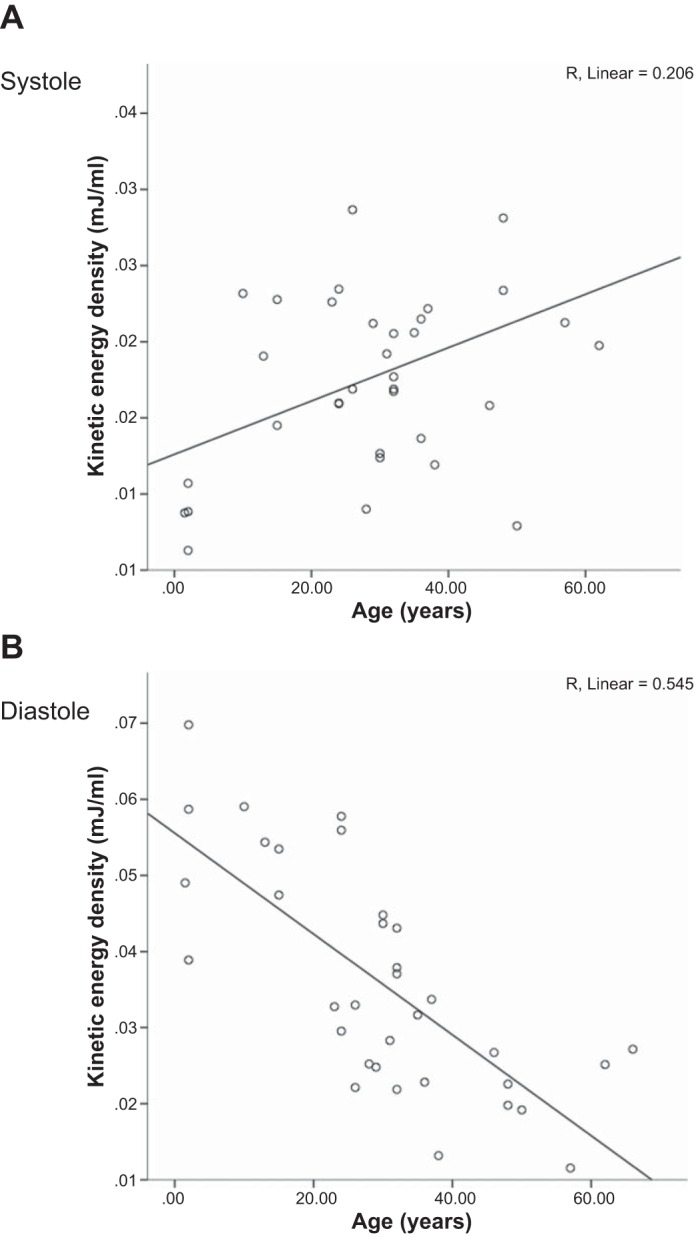
Peak KE in systole (*A*) and early diastole (*B*) for the healthy LV against age. With aging, systolic KE had a mildly positive correlation, whereas diastolic KE had a moderate negative correlation.

**Fig. 4.**
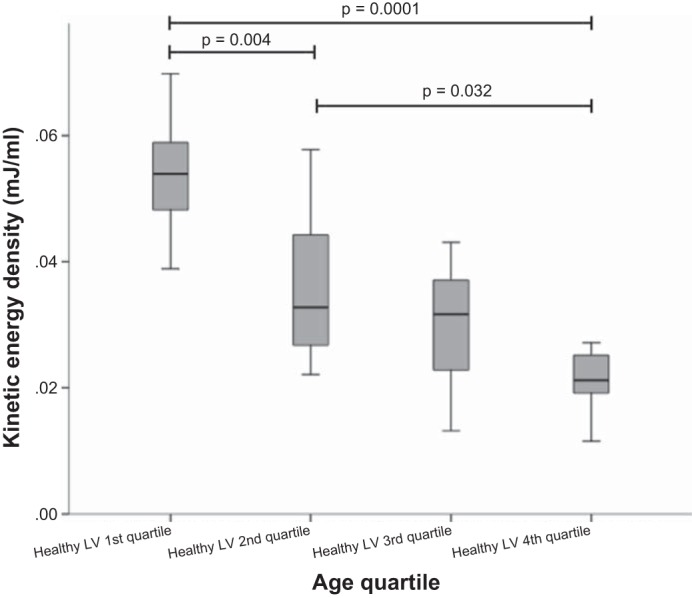
Box and whisker plot of early diastolic KE against age quartile in healthy subjects. Diastolic KE was significantly lower in older adults from the 4th age quartile than children in the 1st age quartile and young adults in the 2nd age quartile.

Changes in intracardiac KE during the cardiac cycle are shown in Fig. 2. Two large peaks were seen in the cardiac cycle of all adults corresponding to systole and early diastole. In eight adults, with the slowest HRs and hence longest R-R intervals, the prospectively ECG gated MRI sequence detected an additional third smaller peak corresponding to late diastole. Children displayed two KE peaks, but the second peak was a monotonic diastolic peak occupying almost all of diastole without a diastasis period. The mean peak and trough values for each age group are shown in Table 3. KE was plotted against age in both systole (Fig. 3*A*) and early diastole (Fig. 3*B*). There was a very weakly positive correlation between systolic KE and increasing age (*R*^2^ = 0.206, *P* = 0.007). However, there were no significant differences between LV outflow tract velocities or ventricular volumes at which peak systolic KE occurred to explain this (Table 4). Peak early diastolic KE showed a negative correlation with age (*R*^2^ = 0.545, *P* < 0.0001). In healthy subjects, there were no differences in mean systolic KE peaks at any age. There were distinct differences in mean early LV diastolic peaks with age (*P* = 0.0001). A progressive fall in mean early diastolic KE peaks occurred between the 1st and 2nd age quartiles (*P* = 0.007) and a second further fall between the 2nd and 4th age quartiles (*P* = 0.025). This is most clearly seen in the box and whisker plots shown in Fig. 4.

**Table 3.** Intracardiac KE

	Systolic KE, mcJ/ml	End-Systolic KE, mcJ/ml	Early Diastolic KE, mcJ/ml	Diastasis KE, mcJ/ml
LV 1st quartile (<16 yr, *n* = 8)	14.3 (±6.6)	3.2 (±2.0)	53.8 (±9.2)*	
LV 2nd quartile (17–32 yr, *n* = 11)	17.9 (±5.7)	2.4 (±0.6)	36.1 (±12.5)†	2.3 (±0.7)
LV 3rd quartile (33–48 yr, *n* = 11)	18.6 (±4.5)	3.1 (±1.3)	29.1 (±9.1)	3.0 (±1.0)
LV 4th quartile (49–64 yr, *n* = 5)	17.8 (±6.3)	2.7 (±1.2)	20.6 (±6.1)	3.6 (±2.1)
*P* value by ANOVA	0.244	0.517	0.0001	0.033
LV dysfunction (28–79 yr, *n* = 10)	12.6 (±5.0)	3.9 (±1.8)	17.1 (±6.3)	7.4 (±4.0)
*P* value (older adults vs. LV dysfunction)	0.033	0.217	0.335	0.051

KE, kinetic energy. *P* values compared with older adults are shown.

* *P* < 0.001; †*P* < 0.05.

**Table 4.** Peak systolic LV outflow tract velocity and peak E wave mitral inflow velocity from four-dimensional flow cardiac magnetic resonance with corresponding ventricular volumes at peak KE

	Peak LV Outflow Tract Velocity, cm/s	Instantaneous Ventricular Volume at Peak Systolic KE, mls	Peak E Wave Velocity, cm/s	Instantaneous Ventricular Volume at Early Peak Diastolic KE, mls
LV 1st quartile (<16 yr, *n* = 8)	134 ± 23	57.4 ± 10.9	106.3 ± 22.6	46.0 ± 10.3
LV 2nd quartile (17–32 yr, *n* = 11)	131 ± 22	67.1 ± 10.2	106.7 ± 29.2	62.9 ± 13.2
LV 3rd quartile (33–48 yr, *n* = 11)	122 ± 20	71.2 ± 20.9	92.1 ± 29.4	68.0 ± 18.8
LV 4th quartile (49–64 yr, *n* = 5)	141 ± 28	71.2 ± 14.0	75.6 ± 16.7	62.7 ± 15.1
*P* value by ANOVA	0.471	0.216	0.098	0.026
*R*^2^ (*P* value)	0.00 (0.99)	0.112 (0.05)	0.139 (0.028)	0.130 (0.03)

P values compared with older adults are shown.

**Fig. 5.**
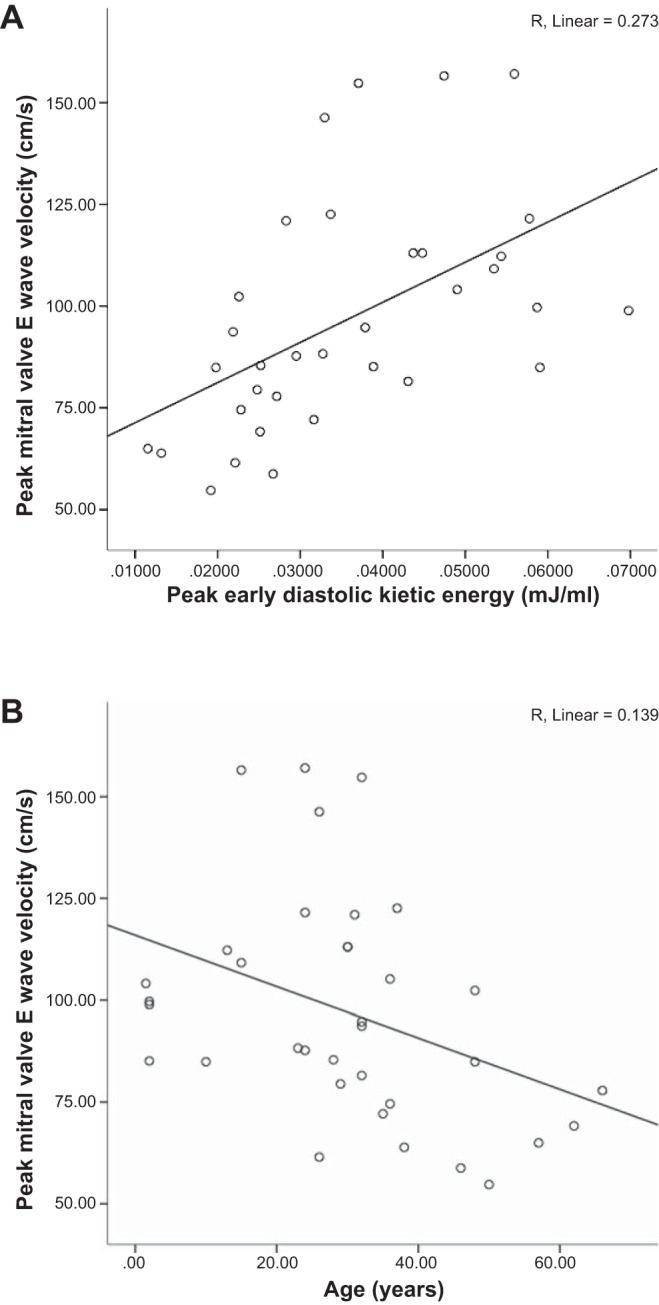
Correlation of mitral valve E wave velocity with peak early diastolic KE (*A*) and age (*B*). There was a moderate correlation between mitral valve E wave velocities and diastolic KE.

Comparison was made between mitral inflow velocities segmented from the 4-D flow and peak early diastolic KE (Table 4 and Fig. 5). Peak mitral valve velocity showed a positive correlation with diastolic early KE (*R*^2^ = 0.273, *P* < 0.002). There was a negative correlation with age (*R*^2^ = 0.139, *P* < 0.028).

There is a potential confounding effect of LV mass and HR on KE. Quartiles were additionally analyzed for indexed LV mass and HR. Using mass indexed to body surface area, a significant decrease in early peak KE between the 1st and 4th index LV mass quartiles was seen (1st quartile: 0.0432 ± 0.0155 mJ/ml vs. 4th quartile: 0.0217 ± 0.006 mJ/ml, *P* = 0.024). Importantly, there were no differences detected among fully grown adults represented by the 2nd, 3rd, and 4th mass quartiles. Peak diastolic KE did not vary significantly between HR quartiles (*P* = 0.051).

**Fig. 6.**
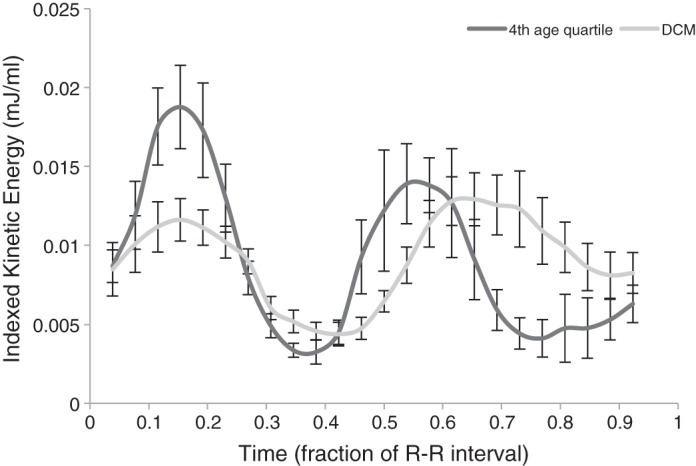
Mean KE of 4th quartile (oldest) adults compared with subjects with LV dysfunction. Subjects with LV dysfunction had KE profiles with a similar diastolic KE peak compared with older adults but with additional changes including a lower systolic KE peak and a higher KE in diastasis.

**Fig. 7.**
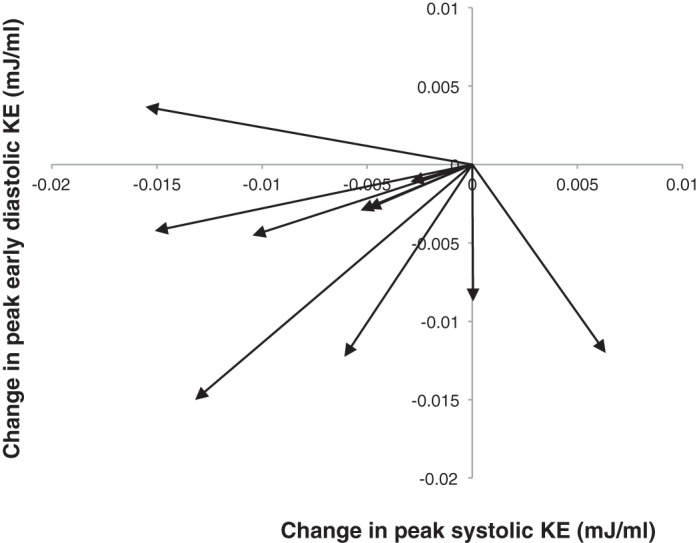
Changes in peak KE during systole and early diastole for subjects with LV dysfunction compared with age- and sex-matched healthy control subjects. The origin point was used as the index for all healthy control subjects. The arrows demonstrate the change in KE for the matched LV dysfunction subjects. Values below the origin represent a decrease in peak early diastolic KE for LV dysfunction subjects compared with healthy control subjects, whereas values to the left of the origin represent a decrease in peak systolic KE.

Patients with LV dysfunction displayed a reduced mean systolic peak (*P* = 0.015; Fig. 6). The early LV diastolic KE peak in the LV dysfunction group was of similar magnitude to older adults (*P* = 0.254), giving an overall broad flattening to the KE profile and an increased KE during diastasis (*P* = 0.029) compared with the older adult group. Age and sex matching of those with LV dysfunction to healthy LV subjects showed that 9 of 10 patients had reduced systolic and diastolic KE values compared with their counterparts. LV dysfunction subjects displayed a lower mean peak systolic KE (0.0132 ± 0.0041 vs. 0.0200 ± 0.0078 mJ/ml, *P* = 0.014) and mean peak diastolic KE (0.0185 ± 0.0064 vs. 0.0245 ± 0.0036 mJ/ml, *P* = 0.01; Fig. 7).

## DISCUSSION

In the present study, we quantified intracardiac KE in the LV over a wide range of ages, specifically comparing the physiological adaptations of healthy aging with the pathological alterations apparent in heart failure. The derived values of KE were of similar magnitude to previously published data despite the use of a different methodology (5). The major findings were a significant fall in early diastolic KE occurring between childhood and early adulthood and a second further significant fall occurring after 40 yr of age. The reduced early diastolic KE in healthy older adults resembled the values seen in those with heart failure. The latter group additionally displayed a broadly flattened intracardiac KE profile.

The compliance of the LV has an important role in efficient ventricular filling (5). Active relaxation of the LV muscle mass during diastole contributes to the suction of blood from the left atrium and pulmonary veins into the ventricle. Decreased ventricular compliance is associated with less favorable filling and diastolic function. During healthy aging, changes in LV morphology occur that have been linked to a decline in diastolic function. Load-independent parameters of cardiac properties show increased LV stiffness (2) that becomes noticeable after 50 yr of age (12). This is due to an accumulation of collagen and increased extracellular matrix cross-linking leading to altered chamber geometry (6) and decreased compliance (2, 20, 32). In our study population, we were unable to measure ventricular stiffness, but there was a small rise in LV wall mass. The main finding was that healthy aging was associated with a fall in peak early diastolic KE. Interestingly, two distinct falls in diastolic KE occurred: a change from a large monotonic KE peak in childhood to an adult profile with a smaller early diastolic peak, with a further decrease in the early diastolic KE peak in late adulthood represented by the 4th quartile, which was aged 49 yr and above. Previously, changes in ventricular compliance have been described after the age of 50 yr (15). Changes in blood flow are extremely sensitive to altered ventricular myocardial properties, and early diastolic KE might appear to represent a noninvasive marker of change.

Interestingly, age-associated increases in cardiac stiffness have been suggested as providing the substrate for heart failure with preserved EF (30). Diastolic KE could be used as a surrogate marker for ventricular compliance, and applying this technique to patients with heart failure with preserved EF could provide additional insights into the pathophysiological mechanisms (26–28).

During healthy aging, preservation of systolic function has been described in the published literature (9). We found similar systolic KE throughout the age cohort in our study. This contrasts to a lower systolic KE in patients with heart failure. These patients additionally displayed an increased energetic baseline during diastasis that may be related to the altered filling mechanisms caused by ventricular dilatation. During diastolic filling, energetic constraints usually limit the maximal growth of the vortex ring that is able to form beneath the atrioventricular valve (13). In the presence of excessive ventricular dilatation, the ring is unable to maintain energetic efficiency while growing to fill the ventricle. This leads to a trailing jet of high-energy dissipation and might be reflected in the elevated KE seen during diastasis in those with established heart failure.

### 

#### Relevance to previous studies.

Early pioneering work in determining KE in humans was performed by Prec et al. (31) using invasive catheterization techniques. Six patients were studied, and the mean total KE produced in one cardiac cycle was 11 mJ for the LV, representing 0.25–2.0% of the external work of the left heart. They concluded that the limitations of their technique might lead to an underestimation of values, particularly under stress. The total KE values were comparable to our 4-D flow study (calculated as the area under the curve). Recent studies (8, 11) performed using CMR have also derived values of a similar magnitude, lending credibility to CMR-based techniques. Our methodology for deriving KE differed slightly from these studies. Carhäll et al. (7, 8) chose a single time point (isovolumetric contraction) and then tracked particles forward and backward through the cardiac cycle to calculate KE. In contrast, we calculated instantaneous KE from the velocity data based on cine segmentations of the ventricle. This was similar to Carlsson et al. (5), differing only in the level of manual segmentation. Both techniques produce values of KE that are very similar and appear equally valid.

These studies looked at the LV and RV in healthy adults only. The novelty of our study is the addition of a broader age range of subjects, including children and a positive control group of patients with LV dysfunction.

An existing study (7) on KE in patients with compensated dilated cardiomyopathy demonstrated preserved stroke volume compared with healthy control subjects. However, the proportion of blood that enters the heart in diastole and is then ejected in the following systolic contraction (direct flow) is significantly reduced. Comparably, we note that our LV dysfunction population also had identical stroke volumes compared with our healthy control group. Although we did not measure direct flow, we were able to demonstrate a significantly reduced peak early diastolic KE value.

Accuracy of the 4-D flow data, particularly velocity, is important in measuring KE. Previous work performed by our group (14–16) has shown the validity of using accelerated 4-D flow sequences to acquire highly temporally undersampled data. Mean bias was only −0.06 m/s with no systematic bias seen on Bland-Altman plots. The 4-D flow data used in this study were meticulously collected. The velocity encoding value was determined using a 2-D PC sequence before the 4-D flow scan to ensure peak velocities were not underestimated. Reassuringly, early diastolic KE correlated with mitral inflow velocities, a finding in keeping with a previous echocardiographic study (40). However, this did not reach significance between the different adult groups. This may be a limitation of the study size.

This study adds important information to our understanding of KE within the heart by virtue of its three-dimensional (3-D) acquisition. Grothues et al. (18) showed CMR data on LV function and dimension to have superior reproducibility compared with echocardiographic measurements. Echocardiography is reliant on flow being directed toward or away from the insonation point. Velocities may be underestimated when using 2-D echocardiography. Application of 3-D techniques allows assessment of the peak velocity within the voxel irrespective of the vector direction. 3-D CMR acquisitions provide a detailed overview of the velocity of blood in three directions, helping to capture the intricacies of flow within the whole heart. This is important, as filling of the LV cavity does not occur solely from the base to apex. Vortexes form below the mitral ring and move apically during healthy diastolic filling (21). Furthermore, a recent study (10) has demonstrated changes in vortex formation with aging. 2-D PC MRI or echocardiography may miss these subtleties or require larger study populations before differences become detectable. Additionally, the mitral valve is elliptical and moves throughout the cardiac cycle (5). It becomes distorted in cardiac disease, such as dilated cardiomyopathy, making accurate plane orientation for 2-D PC flow techniques more technically challenging. 3-D flow echocardiography is currently a tool under development, but this too is limited by the patient's habitus (17).

#### Limitations.

The 4-D flow MRI sequences used prospective ECG triggering, which acquired 90% of the cardiac cycle, missing some of late diastole. This meant that atrial peaks were not evident in all subjects. However, given that the majority of changes seemed to occur in systole and early diastole, we were still able to draw interesting conclusions from the data.

The present study examined age-related changes in comparably large age ranges. More volunteers and further subgroups of age extending toward the older population would allow quantification of when age-related changes tend to manifest themselves. Although the children included in this study were free of cardiovascular disease, it should be noted that they were undergoing a MRI scan for other medically indicated reasons.

This study used CMR data. Therefore, although the results are in keeping with other studies measuring KE in the heart, they were not validated against invasive data.

#### Conclusions.

In summary, we found that a decline in diastolic KE is apparent with age, whereas systolic KE is preserved. These changes may represent a spectrum of physiological deterioration that predisposes toward pathological outcomes. The motion of blood through the heart is highly sensitive to changes in myocardial properties. Flow-derived KE might therefore allow earlier insights into physiological and pathological changes than currently possible.

## GRANTS

The Division of Imaging Sciences receives support as the Centre of Excellence in Medical Engineering (funded by the Wellcome Trust and Engineering and Physical Sciences Research Council, Grant WT 088641/Z/09/Z) as well as the British Heart Foundation (BHF) Centre of Excellence (BHF Award RE/08/03) and BHF New Horizons program (Grant NH/11/5/29058).

The authors acknowledge financial support from the Department of Health via the National Institute for Health Research comprehensive Biomedical Research Centre award to Guy's & St Thomas' National Health Service (NHS) Foundation Trust in partnership with King's College London and King's College Hospital NHS Foundation Trust.

## DISCLOSURES

No conflicts of interest, financial or otherwise, are declared by the author(s).

## AUTHOR CONTRIBUTIONS

Author contributions: J.W., R.C., A.d.V., N.D., E.S., T.S., and R.R. conception and design of research; J.W., R.C., and E.S. performed experiments; J.W., R.C., and A.d.V. analyzed data; J.W., R.C., A.d.V., N.D., E.S., T.S., and R.R. interpreted results of experiments; J.W. prepared figures; J.W. and R.R. drafted manuscript; J.W., R.C., A.d.V., N.D., E.S., T.S., and R.R. edited and revised manuscript; J.W., R.C., A.d.V., N.D., E.S., T.S., and R.R. approved final version of manuscript.
